# Prevalence and Risk Factors of Dry Eye Disease in Association With the Increased Use of Electronic Devices Among University Students in Western Saudi Arabia

**DOI:** 10.7759/cureus.51554

**Published:** 2024-01-02

**Authors:** Noora A Zarban, Omar B Alammari, Saeed Abu Sabah, Nawaf Saleh M Alshamrani, Muath A Alqathanin, Norah A AlRabeeah, Samaher G Basalib

**Affiliations:** 1 Emergency Medicine, King AbdulAziz University, Jeddah, SAU; 2 College of Medicine, King Khalid University Hospital, Riyadh, SAU; 3 Ophthalmology, King Khalid University Hospital, Riyadh, SAU; 4 College of Medicine, Al-Maarefa University, Riyadh, SAU; 5 College of Medicine, Ibn Sina National College for Medical Studies, Jeddah, SAU

**Keywords:** saudi arabia, pediatrics, adults, dry eye disease, ocular surface disease index, osdi, osdi scoring system, osdi questionnaire, prevalence of dry eye symptoms, dry eye disorder

## Abstract

Aim: Dry eye disease (DED) is a prevalent ocular condition that significantly impacts individuals' quality of life and performance. It is charac­terized by the instability of the tear film, which causes ocular surface inflamma­tion and damage that leads to ocular symptoms. However, this study aimed to determine the prevalence of DED and identify associated risk factors among university students in western Saudi Arabia.

Methods: A total of 402 university students participated in this study. The sample size was determined using Raosoft software (Raosoft, Inc., Seattle, WA), considering an estimated student population of 20,000. Data were collected between January and March 2023 through an online questionnaire distributed to the participants. The questionnaire comprised three sections, covering general information, behaviors related to digital device (DD) use, and the validated Arabic version of the Ocular Surface Disease Index (OSDI) questionnaire. OSDI scores were calculated, and the severity of DED was categorized using established cutoff points.

Results: Among the 402 university students who took part in the survey, the majority (63.2%) were aged between 21 and 25 years, with females representing the dominant gender (72.9%). Notably, 90.8% of participants reported using DDs at bedtime. Over 60% of students had been using DDs for more than 10 years, and approximately 61.7% reported having more than six hours of daily screen time. Mobile devices were the most commonly used electronic devices (67.2%), and TikTok emerged as the most frequently used application (35.6%). Based on the OSDI criteria, 21.1% of students had mild DED symptoms, 14.9% had moderate symptoms, and 38.6% had severe symptoms. Hence, the prevalence of students exhibiting positive DED symptoms was 74.6%, while 25.4% were negative.

## Introduction

The Tear Film and Ocular Surface Dry Eye Workshop II (TFOS DEWS II) defines dry eye disease (DED) as "a multifactorial disease of the ocular surface characterized by a loss of homeostasis of the tear film and accompanied by ocular symptoms, in which tear film instability and hyperosmolarity, ocular surface inflammation and damage, and neurosensory abnormalities play etiological roles" [1]. This disease has affected the vision-related quality of life of patients and their career performance [2,3]. Moreover, DED has been found to be the most prevalent chronic ocular condition encountered in ophthalmic clinics, significantly impacting quality of life [4-8]. The prevalence of DED has dramatically increased in recent decades, with global estimates ranging from 5% to 50% [9]. In the United States, it is estimated that 6.8% of the adult population, corresponding to 16.4 million individuals, has been diagnosed with DED based on participant-reported data from the 2013 National Health and Wellness Survey (NHWS) [10]. A hospital-based study from north India reported a prevalence rate of 32%, with the majority of patients categorized as having moderate to severe DED [11]. Multiple studies have found associations between DED and contact lens use, electronic device use, older age, female gender, menopause, certain chronic diseases, and ocular surface diseases [12-17].

The rise of new lifestyles and daily use of technology has raised public health concerns regarding DED. Additionally, exposure to electronic devices starts from a very early age and intensifies during university years. While DED is commonly associated with aging, it is also prevalent among younger populations, including university students. Studies have reported prevalence rates of DED ranging from 5% to 30% among college students in different regions [18]. Recent studies have also revealed a high prevalence of DED among college students worldwide, including North America (10-30%), Europe (10-20%), and Asia (10-30%) [19-21].

The impact of DED on quality of life can be substantial, leading to decreased productivity, increased healthcare utilization, and decreased overall well-being [22]. The economic burden of DED is also significant, with estimated costs exceeding $55 billion annually in the United States alone [23]. However, limited information is available on the prevalence of DED among college students in the Middle East, where cultural and environmental factors may contribute to differences in the prevalence and risk factors of this condition [24].

Therefore, this study aims to investigate the prevalence, risk factors, and impact of DED among university students in western Saudi Arabia. The findings of this study could provide valuable insights into the potential causes and consequences of DED in young adults in this region and inform the development of targeted interventions to prevent or manage this condition in this population.

## Materials and methods

The present investigation represents a cross-sectional study aimed at determining the prevalence, risk factors, and impact of DED among university students in western Saudi Arabia. The minimum sample size was estimated using the Raosoft software (Raosoft, Seattle, WA, USA), and the resulting number was 375 out of a total estimated student population of 20,000. The statistics the Western universities revealed on their official websites in Saudi Arabia [25] were used as the basis for the study. Prior to data collection, ethical approval was secured from the Biomedical Ethics of King Khalid University, Abha, Saudi Arabia (approval number: ECM#2023-703). The data collection team comprised sixth-year medical students and interns under the supervision of an ophthalmology consultant. The data were collected using an online questionnaire (Google Form) and distributed among 402 participants. The survey was carried out from January to March of the year 2023. Participants were requested to complete the electronic survey, which consisted of three sections, each made up of questions related to various aspects of the disease. Informed consent was obtained by ticking the relevant box before starting the survey. The first section of the questionnaire includes general questions, gender, age, marital status, monthly income, and the use of eyeglasses as well as digital devices (DDs). The questions in the second section of the questionnaire are detailed in Table 1.

**Table 1:** The second section of the questionnaire

Behaviors related to the use of digital devices
(1) For how many years have you been using digital devices?
(2) What is your daily screen time in hours?
(3) Which electronic devices do you use most frequently?
(4) Which application do you use most frequently?
(5) Do you prefer studying using papers or digital devices?
(6) Do you take breaks while using digital devices?
(7) How often do you take breaks while using digital devices?
(8) What is the average length of your breaks?
(9) Do you close your eyes intentionally?
(10) Do you adjust the screen brightness for your digital devices?
(11) Have you experienced screen glare?
(12) Do you use an anti-glare filter?
(13) Do you use screen filters?
(14) How bright is your screen?

The third and final section of the questionnaire comprised the Ocular Surface Disease Index (OSDI) questionnaire developed by Schiffman et al. [26]. A valid Arabic version of the questionnaire was used in this study [27]. The OSDI questionnaire consists of 12 items, each with a 5-point Likert scale category ranging from "none of the time" codes with 1 to "all of the time" codes with 4. To calculate the OSDI score, the sum of scores was multiplied by 25 and then divided by the number of answered questions. The total score ranges from 0 to 100 points, with higher scores indicating greater disability. The cutoff point to determine the severity of DED was based on the study published by Aberame et al. [28], wherein mild (13-22 points), moderate (23-32 points), and severe (33-100 points) constituted the severity levels. Based on these cutoff points, university students were considered to have positive symptoms of DED if they scored ≥13 points; scores below 13 points were considered negative for DED symptoms.

Statistical analysis

Categorical data were shown as frequency and proportion (%). Continuous data were displayed as mean and standard deviation. The relationship between DED symptoms among the socio-demographic characteristics and the behavior of university students when using DDs has been conducted using the Chi-square test. Achieved significant results were then gathered in a multivariate regression model to determine the significant independent predictor associated with the symptoms of DED with a corresponding odds ratio as well as 95% CI. Statistical significance was set to P<0.05. The data were computed and analyzed using the Statistical Package for the Social Sciences (IBM SPSS Statistics for Windows, IBM Corp., Version 26.0, Armonk, NY).

## Results

Four hundred and two university students completed the survey. As seen in Table 2, the most common age group was 21 to 25 years (63.2%), with females being dominant (72.9%). Most of the students were single (73.4%), and 62.2% earned at least less than 5,000 SAR per month. Nearly all (90.8%) were using DDs at bedtime. The prevalence of students who use eyeglasses was 47%, and the most common reason was due to medical eye conditions (78.8%).

**Table 2:** Socio-demographic characteristics of the university students (n=402)

Study variables	N (%)
Age group	
≤20 years	82 (20.4%)
21-25 years	254 (63.2%)
>25 years	66 (16.4%)
Gender	
Male	109 (27.1%)
Female	293 (72.9%)
Marital status	
Single	295 (73.4%)
Married	86 (21.4%)
Widowed	14 (03.5%)
Divorced	07 (01.7%)
Monthly income (SAR)	
<5,000	250 (62.2%)
5,000-10,000	96 (23.9%)
>10,000	56 (13.9%)
Using digital devices at bedtime	
Yes	365 (90.8%)
No	37 (09.2%)
Do you use eyeglasses?	
Yes	189 (47.0%)
No	213 (53.0%)
If yes, why do you use eyeglasses?^(n=189)^	
Medically	149 (78.8%)
While using electronic devices	40 (21.2%)

In Tables 3-4, approximately 60.9% were using DDs for more than 10 years. Students who had more than six hours of daily screen time constitute 61.7%. The most common electronic device used was mobile (67.2%), while the most commonly used application was TikTok (35.6%). Nearly three-quarters (73.9%) were using DDs for studying. Students who took breaks while using the DD were 60.2%. Of them, 41% took breaks every hour, with an average of 5 to 10 minutes (36%). Students who close their eyes intentionally while using DDs constitute 56.5%, while those who adjust screen brightness were 83.3%. Exposure to screen glare was reported by 51%, and those who use an anti-glare were 23.9%. Approximately two-thirds (66.7%) were using DDs with less than 40 cm distance between the eyes and the screen. Only 26.4% were using screen filters. A great proportion of university students (56%) set their DDs to bright screens, and a similar proportion (50.7%) used bright room lighting when using electronic devices.

**Table 3:** Behavior of university students in using DDs (n=402) DDs: digital devices

Statement	N (%)
The number of years using DDs?	
<5 years	25 (06.2%)
5-10 years	132 (32.8%)
>10 years	245 (60.9%)
Daily screen time	
<3 hours	22 (05.5%)
3-6 hours	132 (32.8%)
>6 hours	248 (61.7%)
Which electronic devices are you using the most?	
Mobile	270 (67.2%)
Computer laptop	43 (10.7%)
TV	13 (03.2%)
Digital gaming devices (e.g., PlayStation)	10 (02.5%)
Tablet/iPad	66 (16.4%)
Which application are you using the most?	
TikTok	143 (35.6%)
Snapchat	67 (16.7%)
Instagram	51 (12.7%)
Twitter	44 (10.9%)
Applications for studying (ex: notability/one note/java, etc.)	63 (15.7%)
Video Games	34 (08.5%)
How do you study?	
Digital (iPad/Laptop)	297 (73.9%)
Papers	105 (26.1%)
Do you take breaks while using DDs?	
Yes	242 (60.2%)
No	160 (39.8%)
How often do you take breaks while using the digital device?^(n=261)^	
Every 30 minutes	71 (27.2%)
Every hour	107 (41.0%)
More than 1 hour	83 (31.8%)
What is the average length of the breaks?^(n=261)^	
<5 minutes	45 (17.2%)
5-10 minutes	94 (36.0%)
10-15 minutes	61 (23.4%)
>15 minutes	61 (23.4%)
Do you close your eyes intentionally?	
Yes	227 (56.5%)
No	175 (43.5%)
Do you adjust the screen brightness for your DDs?	
Yes	335 (83.3%)
No	67 (16.7%)
Exposure to screen glare?	
Yes	205 (51.0%)
No	197 (49.0%)
Do you use an anti-glare filter?	
Yes	96 (23.9%)
No	306 (76.1%)

**Table 4:** Behavior of university students in using DDs (n=402) (cont'd) DDs: digital devices

While using DDs, the distance between my eyes and the screen is approximate	
<40 cm (less than an arm's length away)	268 (66.7%)
Between 40cm-76cm (about an arm's length away)	108 (26.9%)
>76 cm (more than an arm's length away)	26 (06.5%)
Do you use screen filters?	
Yes	106 (26.4%)
No	296 (73.6%)
How is your screen bright?	
Very bright	46 (11.4%)
Bright	225 (56.0%)
Dim	131 (32.6%)
How good is the room lighting when you use electronic devices?	
Very bright	30 (07.5%)
Bright	204 (50.7%)
Dim	130 (32.3%)
Very dim	38 (09.5%)

**Figure 1:**
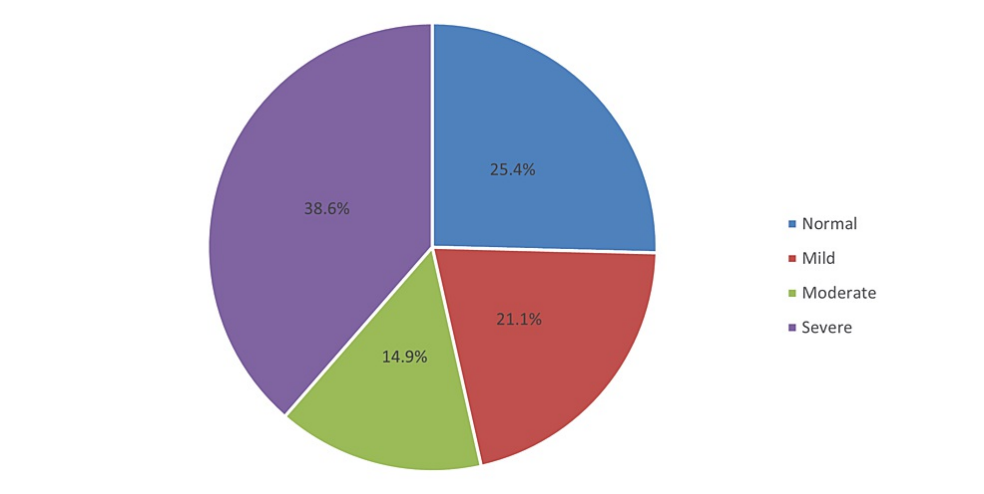
Severity of dry eye disease

**Figure 2:**
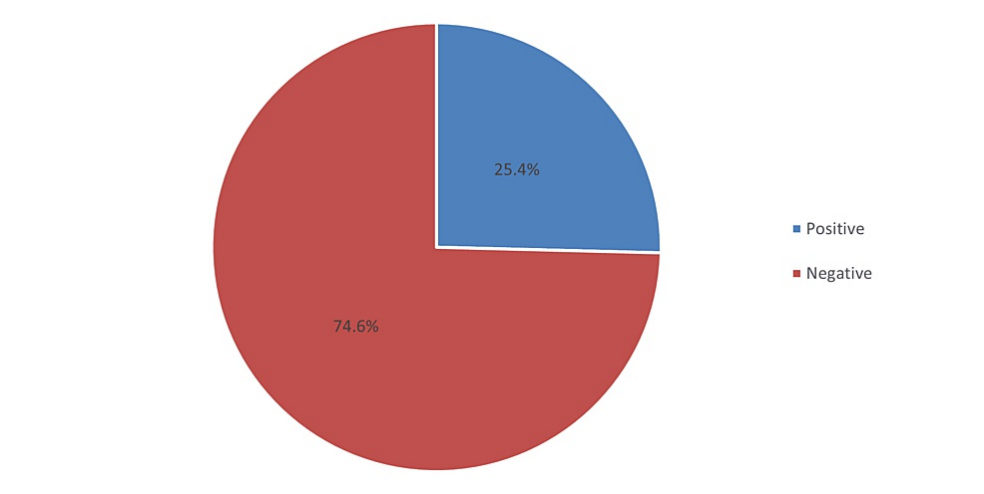
Prevalence of dry eye symptoms

Regarding the prevalence of DED (Table 5), the total mean OSDI score was 30.4 (SD 23.6). Based on the given criteria, mild, moderate, and severe levels constitute 21.1%, 14.9%, and 38.6%, respectively (see Figure 1). Accordingly, the prevalence of students with positive symptoms of DED constituted 74.6%, and the rest were negative (25.4%) (see Figure 2).

**Table 5:** Prevalence of dry eye disease using Ocular Surface Disease Index (OSDI) (n=402)

Variables	N (%)
Total OSDI score (mean ± SD)	30.4 ± 23.6
Severity of dry eye disease	
Normal (score 0-12)	102 (25.4%)
Mild (score 13-22)	85 (21.1%)
Moderate (score 23-32)	60 (14.9%)
Severe (score 33-100)	155 (38.6%)
Symptoms of dry eye disease	
Positive (score ≥13)	300 (74.6%)
Negative (score <13)	102 (25.4%)

When measuring the relationship between the symptoms of DED among the socio-demographic characteristics and the behavior of the students when using DDs (Tables 6-7), it was found that the prevalence of positive DED students was significantly more common among those who were using eyeglasses (p<0.001), using of TikTok app on a DD (p=0.014), closing of eyes intentionally when using a DD (p<0.001) and exposure to screen glare (p=0.006).

**Table 6:** Relationship between the symptoms of DED among the socio-demographic characteristics and the behavior of university students when using DDs (n=402) § P-value has been calculated using the Chi-square test. ** Significant at p<0.05 level DED: dry eye disease, DD: digital devices

Factor	Symptoms of DED		P-value^§^
	Positive N (%)^(n=300)^	Negative N (%)^(n=102)^	
Age group			
≤20 years	59 (19.7%)	23 (22.5%)	0.756
21-25 years	190 (63.3%)	64 (62.7%)	
>25 years	51 (17.0%)	15 (14.7%)	
Gender			
Male	80 (26.7%)	29 (28.4%)	0.729
Female	220 (73.3%)	73 (71.6%)	
Using DDs at bedtime			
Yes	274 (91.3%)	91 (89.2%)	0.523
No	26 (08.7%)	11 (10.8%)	
Do you use eyeglasses?			
Yes	158 (52.7%)	31 (30.4%)	<0.001**
No	142 (47.3%)	71 (69.6%)	
The number of years using DDs?			
≤10 years	110 (36.7%)	47 (46.1%)	0.092
>10 years	190 (63.3%)	55 (53.9%)	
Daily screen time			
≤6 hours	112 (37.3%)	42 (41.2%)	0.490
>6 hours	188 (62.7%)	60 (58.8%)	
Which electronic devices are you using the most?			
Mobile	200 (66.7%)	70 (68.6%)	0.639
Computer Laptop	33 (11.0%)	10 (09.8%)	
TV	12 (04.0%)	01 (01.0%)	
Digital gaming devices	07 (02.3%)	03 (02.9%)	
Tablet/iPad	48 (16.0%)	18 (17.9%)	
Which application are you using the most?			
TikTok	117 (39.0%)	26 (25.5%)	0.014**
Snapchat	54 (18.0%)	13 (12.7%)	
Instagram	34 (11.3%)	17 (16.7%)	
Twitter	28 (09.3%)	16 (15.7%)	
Application for studying	40 (13.3%)	23 (22.5%)	
Video games	27 (09.0%)	07 (06.9%)	
How do you study?			
Digital (iPad/Laptop)	223 (74.3%)	74 (72.5%)	0.723
Papers	77 (25.7%)	28 (27.5%)	
Do you take breaks while using DDs?			
Yes	186 (62.0%)	56 (54.9%)	0.206
No	114 (38.0%)	46 (45.1%)	
Do you close your eyes intentionally?			
Yes	185 (61.7%)	42 (41.2%)	<0.001**
No	115 (38.3%)	60 (58.8%)	

**Table 7:** Relationship between the symptoms of DED among the socio-demographic characteristics and the behavior of university students when using DDs (n=402) (cont'd) § P-value has been calculated using the Chi-square test. ** Significant at p<0.05 level. DED: dry eye disease, DD: digital devices

Factor	Symptoms of DED		P-value ^§^
	Positive N (%) ^(n=300)^	Negative N (%) ^(n=102)^	
Do you adjust the screen brightness for your DDs?			
Yes	247 (82.3%)	88 (86.3%)	0.356
No	53 (17.7%)	14 (13.7%)	
Exposure to screen glare?			
Yes	165 (55.0%)	40 (39.2%)	0.006 **
No	135 (45.0%)	62 (60.8%)	
Do you use an anti-glare filter?			
Yes	78 (26.0%)	18 (17.6%)	0.087
No	222 (74.0%)	84 (82.4%)	
While using DDs, the distance between my eyes and the screen is approximate			
<40 cm (less than an arm's length away)	205 (68.3%)	63 (61.8%)	0.224
≥40cm (about an arm's length away)	95 (31.7%)	39 (38.2%)	
Do you use screen filters?			
Yes	85 (28.3%)	21 (20.6%)	0.125
No	215 (71.7%)	81 (79.4%)	
How is your screen bright?			
Very bright/Bright	208 (69.3%)	63 (61.8%)	0.159
Dim	92 (30.7%)	39 (38.2%)	
How good is the room lighting when you use electronic devices?			
Very bright/Bright	171 (57.0%)	63 (61.8%)	0.399
Dim/Very dim	129 (43.0%)	39 (38.2%)	

When conducting a multivariate regression analysis (Table 8), it was observed that eyeglasses, intentionally closing eyes when using a DD, and exposure to screen glare were identified as the independent significant predictors of positive DED. This further suggests that compared to students who were not using eyeglasses, students who used eyeglasses were at a 2.3 increased risk of having DED symptoms (AOR=2.324; 95% CI=1.413-3.823; p=0.001). Students who close their eyes intentionally while using DDs were 1.99 times more likely to be associated with having DED symptoms (AOR=1.997; 95% CI=1.231-3.239; p=0.005). Also, students who were exposed to screen glare were predicted to increase the risk of having DED symptoms by at least 1.69-fold higher (AOR=1.693; 95% CI=1.044-2.747; p=0.033). However, the type of application being used when using electronic devices was not predicted to increase the risk of having DED symptoms after adjustment to a regression model (p>0.05).

**Table 8:** Multivariate regression analysis for the significant independent predictors that influence positive DED symptoms (n=402) AOR: adjusted odds ratio; CI: confidence interval, DED: dry eye disease ** Significant at p<0.05 level

Factor	AOR	95% CI	P-value
Do you use eyeglasses?			
No	Ref		
Yes	2.324	1.413-3.823	0.001 **
Do you close your eyes intentionally?			
No	Ref		
Yes	1.997	1.231-3.239	0.005 **
Exposure to screen glare?			
No	Ref		
Yes	1.693	1.044-2.747	0.033 **
Which application are you using the most?			
TikTok	Ref		
Snapchat	1.045	0.399-2.738	0.928
Instagram	0.919	0.319-2.645	0.876
Twitter	2.152	0.757-6.122	0.151
Application for studying	2.050	0.709-5.930	0.185
Video games	2.661	0.969-7.310	0.058

## Discussion

This study quantifies the prevalence and risk factors for DED and determines if there is an existing link with using electronic devices among university students. Using the OSDI questionnaire, the prevalence of DED in this study was 74.6%, consisting of mild (21.1%), moderate (14.9%), and severe (38.6%) levels (mean OSDI score: 30.4; SD 23.6). Consistent with our findings, a study published by Alrabghi et al. [29] found a prevalence of DED of 74.9%, with severe cases detected in 30.4%. This has been corroborated by the study of Choi et al. [30], with a prevalence of 78.1%, compromising mild, moderate, and severe cases detected in 25.8%, 18.7%, and 33.5%, respectively. However, in Malaysia [31], a relatively higher incidence of DED was noted among undergraduate medical students, with an incidence rate of 92.1%. In contrast, Supiyaphun et al. [32] documented a lower incidence of DED among university students, at 8.15%, and severe cases were 5.5% only. The prevalence of DED varies according to the region and the target population. However, evidence suggests that university students may have been associated with a high prevalence of DED as compared to the general population [33].

Data from our study suggest that the use of eyeglasses, intentionally closing eyes, and exposure to screen glare were the significant dependent risk factors for DED. These findings are comparable to the study of Altinbas et al. [34]. According to reports, they found a significant relationship between OSDI scores according to the indoor environmental condition in a computer, with using a computer in a dim environment and higher line of sight associated with higher OSDI scores. Similarly, Abdulmannan et al. [35] noted that students wearing contact lenses were at higher risk for developing DED. However, a study by John et al. [31] found no significant factors with positive findings DED by subjective and objective assessment and duration of video display terminal usage. In our study, we also achieved insignificant association between the symptoms of DED in terms of age, gender, use of the DD at bedtime, number of years using a DD, the most common electronic device being used, method used during study, taking breaks while using DDs, adjustment of screen brightness, use of anti-glare filter, distance of DD between eyes and the screen, use of screen filters, and the level of brightness for screen and room lightings (p>0.05).

The behavior when using DD could increase the DED-related symptoms such that the longer the use of DD, the greater the risk for DED symptoms. Consistent with this scenario, several studies documented an association between prolonged use of electronic devices and DED [29,30,32,36,37]. However, this is not the case in a study by Aljammaz et al. [38], as they found no significant association between DED and the number of hours in front of the screen device, along with age, family history of DED, and history of corrective eye surgery. In our study, although we found no significant relationship between screen time and DED symptoms, the excessive usage of DD was evidently seen among our students. In Jordan [37], most medical students (98.6%) use electronic devices before bedtime, which could lead to poor sleep quality and greatly influence the incidence of DED.

Incidentally, Ezinne et al. [39] documented that symptomatic DED had direct associations with the lack of education about dry eye, use of the reading mode for computer, refractive error, previous systemic medication, and average visual display unit use per day. In our study, however, many students were seen to have a lack of education about the safe usage of electronic gadgets. For example, 83.3% used to adjust the screen brightness of their DD, with more than half (51%) being exposed to screen glare. Despite this scenario, some students use methods to protect their eye health, including the used anti-glare (23.9%), screen filters (26.4%), or bright room lighting (50.7%). It is necessary to raise awareness about the hazardous effects of the prolonged use of DDs among our youth. Safe and daily screen time usage should be periodically promoted during the school curriculum to prevent ocular health-related diseases among university students.

Limitations

The generalization of this study was subjected to some limitations. Gender distribution was not equally collected; thus, we cannot generalize the comparison of DED symptoms between males and females. Also, being cross-sectional is prone to disadvantages, including cause-and-effect relationships, and prone to bias. In addition, a survey is prone to bias answering as some participants might not be truthful with their answers to the questions.

Recommendations

Further research on this topic is recommended, probably at a national level which can generate a bigger sample size. Hence, this may give us better insights into the prevalence of DED and its association with the electronic devices used among university students. We also advocate health education awareness about the harmful effects of excessive usage of DDs, which promotes daily screen time appropriate to age.

## Conclusions

The study conducted among university students in western Saudi Arabia concludes that DED symptoms were highly prevalent among them, with a rate of 74.6%. This finding is consistent with several other studies conducted on this particular group. Additionally, the research revealed a significant correlation between the use of electronic devices and lower OSDI scores. This suggests that there may be changes in the factors influencing the prevalence of DED, which may require further investigation through new studies.
